# A Pharmacovigilance Study of Antihypertensive Medicines at a South Delhi Hospital

**DOI:** 10.4103/0250-474X.56018

**Published:** 2009

**Authors:** A. Hussain, M. Aqil, M. S. Alam, M. R. Khan, P. Kapur, K. K. Pillai

**Affiliations:** Faculty of Pharmacy, Jamia Hamdard (Hamdard University), M. B. Road, New Delhi-110 062, India; 1 Majeedia Hospital, Jamia Hamdard Campus (Hamdard University), New Delhi-110 062, India

**Keywords:** Pharmacovigilance, adverse drug reactions, antihypertensive, beta-blockers

## Abstract

The aim of the present study was to monitor adverse drug reactions associated with antihypertensive drugs. The study was conducted in medicine out patient department of 150-bed Majeedia Hospital at Hamdard University Campus in New Delhi. The study was conducted by way of one to one patient interview by a registered pharmacist using a questionnaire-based Adverse Drug Reaction Monitoring Form drafted according to the World Health Organisation Monitoring Guidelines. A total of 34 adverse drug reactions were observed in 250 hypertensive patients during the four month study. A high percentage of adverse drug reactions occurred in middle aged and female patients. Of the 34 adverse drug reactions, 18 (52.9%) were mild, 14 (41.2%) moderate and only 2 (5.8%) were classified as severe. Combination therapy was associated with significantly high occurrence (P < 0.05) of adverse drug reactions, with a total of 21 (61.8%) as compared to monotherapy (n=13, 38.2%). Cardiovascular adverse drug reactions constituted a major component, followed by gastrointestinal and respiratory complaints. Beta-blockers were the drug category associated with majority of adverse drug reactions, followed by angiotensin-converting enzyme inhibitors and calcium channel blockers. The above pharmacovigilance study presents the adverse drug reaction profile of antihypertensive medicines prescribed in our University Teaching Hospital. It was concluded that calcium channel blockers were the most frequently prescribed drug category but beta blockers were associated with higher frequency of adverse drug reactions.

According to the World Health Organization (WHO) definition, an adverse drug reaction (ADR) is ‘a response to a drug that is noxious and unintended and occurs at doses normally used in human for the prophylaxis, diagnosis, and treatment of disease, or for modification of physiological function’[1]. ADR can also be defined as ‘an appreciably harmful or unpleasant reaction, resulting from an intervention related to the use of a medicinal product, which predicts hazard from future administration and warrants prevention or specific treatment, or alteration of the dosage regimen, or withdrawal of the product’[2].

Adverse drug reactions (ADRs) are considered among the leading causes of morbidity and mortality. Around 6% of hospital admissions are estimated to be due to ADRs and about 6-15% of hospitalized patients experience a serious ADR[3]. When the Food and Drug Administration (FDA) approves a new drug for marketing, its complete adverse advents profile may not be known because of the limitation of pre- approval clinical trials. Typically, clinical trials for new drugs are of short duration and are conducted in populations that number up to 5000, therefore, the most common dose related ADRs are usually detected in the pre-marketing phase while ADRs which are rare and those detected on long term use are not. A case in point is the development of brownish blue pigmentation of nails of a patient on atenolol for 3 years. Another patient on amlodipine for 8 years developed Schamberg's like purpuric pigmentation[4].

Since most trials exclude the elderly, children, pregnant women, patients with multiple diseases, and those on medication suspected of interaction with the study drug, the study population may not be true representative of the real world where the drug is eventually used[5]. Hence, there is a need to monitor the safety profile of all the medications on continuous basis and to review their therapeutic rationale in the light of add on information emanating out of the pharmacovigilance activities. Monitoring of ADRs is even more important in case of chronic ailments such as hypertension. More often than not, hypertension is an asymptomatic disorder and requires long term therapy predisposing to adverse drug events[5].

Pharmacovigilance studies for monitoring ADRs related to antihypertensive agents have been previously conducted by many workers in different parts of the world[6–8]. Monitoring of ADRs in India is in its infancy[9]. A study conducted in the Indian capital reports that 22.3% of the patients experienced ADRs[10]. Another report on ADR monitoring in northern India mentions that 5.9% of all visits to the medical department are drug related, and ADRs accounted for 45% of events[11].

This study is aimed to evaluate the incidence of ADRs in patients receiving anti-hypertensive agents in our university teaching hospital. The ADR reporting is primarily based on drug categories, but sex, age, and weight have also been included as explanatory variables. The present work was an open, non-comparative, observational study to monitor ADRs associated with antihypertensive medicines in our university teaching hospital. The data was recorded on a questionnaire based Adverse Drug Reaction Monitoring (ADRM) form drafted according to WHO monitoring guidelines, which included data related to patient demographics (age, sex, height, weight, body mass index (BMI)), past medical history, present drug treatment, description, assessment and treatment of ADR.

The study protocol was approved by Jamia Hamdard University Institutional Review Board (Approval letter No 02/ 06, JH-IRB dated February 16, 2006). The study was conducted between February to May 2006 by a registered pharmacist attending the medicine OPD on a daily basis. An informed consent form was taken from the patients participating in the study. All newly diagnosed and old patients receiving antihypertensive medications irrespective of age and sex were included in the study. All mentally compromised or unconscious patients and patients unable to respond to verbal questions were excluded from the study. All drug-related adverse events were evaluated according to the “WHO Probability Assessment Scale”[12] (Table 1). In calculating the ADRs associated with a specific drug category, a minimum of 6 prescriptions were considered for significant result. Student's *t* test was used for statistical analysis at P<0.05 using Graph Pad Instat software Version 3.06.

**TABLE 1:** WHO PROBABILITY ASSESSMENT (CAUSALITY ASSESSMENT) SCALE FOR ADVERSE DRUG REACTIONS

Category	Description
1. Certain	A clinical event, including laboratory test abnormality, that occurs in a plausible time in relation to drug administration and which cannot be explained by concurrent disease or other drugs or chemicals. The response to withdrawal of the drug (dechallenge) should be clinically plausible. The event must be definitive pharmacologically or phenomenologically, using a satisfactory rechallenge procedure, if necessary.
2. Probable	A clinical event, including a laboratory test abnormality with a reasonable time relation to administration of drug, unlikely to be attributed to concurrent disease or other drug or chemical and which follows a clinically reasonable response on withdrawal (dechallenge). Rechallenge information is not required to fulfil this definition.
3. Possible	A clinical event, including laboratory test abnormality, with a reasonable time relation to administration of the drug, but which could also be explained by concurrent disease or other drug or chemicals.
4. Unlikely	A clinical event including a laboratory test abnormality, which makes a causal relation improbable, and in which other drugs, chemical or underlying diseases provide plausible explanations.
5. Conditional/unclassified	A clinical event, including a laboratory test abnormality, reported as an adverse reaction, about which more data are essential for a proper assessment, or the additional data are being examined.
6. Inaccessible/unclassifiable	A report suggesting an adverse reaction that cannot be judged because information is insufficient or contradictory and cannot be supplemented or verified.

As per WHO causality assessment criteria, ADR were classified as Certain, Probable, Possible, Unlikely, Conditional/unclassified and Inaccessible/unclassifiable.

A total of 34 ADRs were observed in 250 hypertensive patients (106 male and 144 female) during the four month of study with a mean age of 51.52±12.1; mean BMI of 41.52±13.9 kg/m^2^ (Table 2). A higher percentage of ADRs occurred in females 20 (58.8%) than males 14 (41.2%). A total of 12 ADRs (35.3%) were observed in the patient age group of 41-50 y, followed by 9 (26.5%) in 51-60 y, 5 (14.7%) in 61-70 y, 4 in 31-40 y, 3 ADRs in >70 y and only 1 (2.9%) ADR in 20-30 y age group. Of the 34 ADRs, 18 (52.9%) were mild, 14 (41.2%) moderate and only 2 (5.8%) was classified as severe (one patient developed severe bronchospasm, syncope and generalized weakness with metoprolol (100 mg) and another developed severe hypotension (B.P. 90/59 mmHg) with atenolol (50 mg). Combination therapy was associated with significantly high occurrence (*P* < 0.05) of ADRs, with a total of 21 (61.8%) as compared to monotherapy (n=13, 38.2%). Among the organ systems affected, cardiovascular ADRs constituted a major component (35.3%), followed by gastrointestinal complaints (20.6%) and respiratory complaints (11.8). On the causality scale of WHO, 16 (47.1%) ADRs were classified possible, 12 (35.5%) probable, 4 (11.8%) unlikely, 1 (2.9%) certain, and 1 (2.9%) could not be categorized (unclassifiable). Among the 250 patients, 131 patients were treated with beta-blockers including atenolol (n= 68), nebivolol (n=32) and metoprolol (n=31). Of these 11 (8.4%) patients experienced ADRs viz., hypotension (2.9%), giddiness (2.9%), headache (1.4%) and bradycardia (1.4%) with atenolol; impotence (3.2%), bronchospasm (6.4%) and irritation over whole body (3.2%) with metoprolol; and pedal edema (3.1%) with nebivolol. A total of 69 patients received treatment with ACE inhibitors comprising of ramipril (n=61) and enalapril (n=8). Among these a total of 5 (7.2%) patients experienced ADRs. Dry cough was the only ADR observed with ACE inhibitors with 12.5% and 6.5% of complaints due to enalapril and ramipril, respectively. Calcium channel blockers (CCBs) were administered to 138 patients. Among these 9 (6.5%) patients experienced ADRs, including pedal oedema (2.3%), headache (1.5%), swelling of the face (0.8%), general oedema (0.8%), and giddiness (0.8%) with amlodipine and bradycardia (16.1%) with nifidipine (Table 3).

**TABLE 2:** DEMOGRAPHIC CHARACTERISTICS OF HYPERTENSIVE PATIENTS

Patient characteristics	Mean±standard deviation
Age (years)	51.52±12.10
Weight (kg)	67.78±12.45
Height (cm)	157.86±11.18
Body mass index (kg/m^2^)	41.52±13.90

Demographic characteristics of study patients; out of the total 250, males were 106 and females were 144.

**TABLE 3:** ADVERSE DRUG REACTIONS AND THERAPEUTICS CLASS OF SUSPECTED MEDICATION

Drugs	Adverse events experienced	Total No of patients with ADRs/No of Patients receiving drugs	% ADRs
Calcium channel blockers			
Amlodopine	Pedal edema -3, Oedema - 1	8/132	6.1%
	Headache, abdominal pain - 2		
	Swelling of face - 1		
	Giddiness -1		
Nefidipine	Bradycardia -1	1/6	16.7%
Total		9/138	**6.5%**
Beta-blockers			
Atenolol	Hypotension - 2	6/68	8.8%
	Giddiness -2		
	Headache -1		
	Bradycardia -1		
Metoprolol	Impotence -1	4/31	12.9%
	Bronchospasm -2		
	Irritation over whole body -1		
Nebivolol	Pedal edema - 1	1/32	3.1%
Total		11/131	**8.4%**
ACE Inhibiters			
Ramipril	Dry cough -4	4/61	6.5%
Enalapril	Dry cough -1	1/8	12.5%
Total		5/69	**7.2%**
Others			
Telmisartan	Dry cough -1	1/10	
Frusemide	Hypotension -1	2/28	
	Bradycardia -1		
Hydrochloro-thiazide	Muscle cramps-1,	4/18	
	Headache-1		
	Vertigo -1		
	Pain in legs -1		
Prazosin	Headache-1	2/6	
	Postural hypotension -1		
Total		9/62	**14.5%**

ADRs and therapeutics class of the possible causative medication, calcium channel blockers 9 ADRs out of 138 prescription drugs, beta-blockers, 11 ADRs out of 131 prescription drugs, ACE inhibiters 9 ADRs out of 69 prescription drugs and others 9 ADRs out of 62 prescription drugs.

In our study, the female hypertensive population was found to be more susceptible to ADRs than the male one. Most of the ADRs were mild or moderate only a couple of cases of ADRs were severe as the patients suffered from severe hypotension and needed to be hospitalized. The result confirms previous reports that the occurrence of ADRs is on the higher side in females[13–15]. Though according to a recent survey, the overall tolerability of low to moderate dose antihypertensive medicines is likely to be similar in men and women[3,16,17]. As expected, combination therapy was associated with higher number of ADRs as compared to monotherapy. Amlodipine and atenolol combination therapy leads to greater risk of ADRs than the monotherapy as reported earlier[6,18,19]. In this study we found that CCBs were the commonest group of drugs prescribed, though beta-blockers and ACE inhibitors were associated with higher incidences of ADRs. Our findings corroborate the results of previous studies which mention beta-blockers as the drug category most often implicated with ADRs[6,20]. Hence, there is a need to review the status of beta-blockers in management of hypertension. Recent prescribing patterns also suggest preferential use of CCBs (31.7%), over beta blockers (7.5%)[21].

The ADRs associated with CVS were found to be most frequent in our study followed by gastrointestinal ADRs (abdominal pain, constipation and diarrhoea). This is supported by previous studies which report gastrointestinal ADRs among the top three ADRs[3,15,18]. The shortcomings of this study are the absence of a control group to which we could have related the incidence of ADRs. Also the sample size was small and special groups like pregnant women, mentally challenged patients and drug addicts were not included in the study.

The present work is a part of ongoing pharmacovigilance program in our university hospital. Beta-blockers were most frequently associated with ADRs, followed by angiotensin-converting enzyme inhibitors and calcium channel blockers. The results of the above study would be useful for the physicians in rational selection of drug therapy for treatment of hypertensive patients. The present data suggest that the ADR monitoring needs to be done in hospital settings continuously so that untoward effect caused by different medicines can be identified and documented.

## References

[1] World Health Organization International drug monitoring: The role of national centers. Tech Rep Ser WHO 1972, No. 498.

[2] Edwards IR, Aronson JK (2000). Adverse drug reactions: Definitions, diagnosis, and management. Lancet.

[3] Jose J, Rao GM (2006). Pattern of adverse drug reactions notified by spontaneous reporting in an Indian tertiary care teaching hospital. Pharmacol Res.

[4] Upadhayai JB, Nangia AK, Mukhija RD, Misra M, Mohan L, Singh KK (2006). Cutaneous reactions due to antihypertensive drugs. Indian J Dermatol.

[5] Ahmad SR (2003). Adverse drug event monitoring at the food and drug administration. J Gen Intern Med.

[6] Olsen H, Klemetsrud T, Stokke HP, Tretlis T, Westheim A (1999). Adverse drug reactions in current antihypertensive therapy: A general practice survey of 2586 patients in Norway. Blood Press.

[7] Wallander MA, Dimenas E, Svardsudd K, Wiklund I (1991). Evaluation of three methods of symptom reporting in a clinical trial of felodipine. Eur J Clin Pharmacol.

[8] Riley J, Wilton LV, Shakir SA (2002). A post marketing observational study to assess the safety of mibefradil in the community of England. Int J Clin Pharmacol Ther.

[9] Dhikav V, Singh S, Anand KS (2004). Adverse drug reaction monitoring in India. J Indian Acad Clin Med.

[10] Parthasarathi G, Olsson S, Parthasarathi G, Nyfort-Hansen K, Nahata MC (2004). Adverse drug reactions. A textbook of clinical pharmacy practice.

[11] Garg KC, Singhal KC, Kumar S (1993). Monitoring the adverse profile of atenolol a collaborative study. Indian J Physiol Pharmacol.

[12] Uppsala Monitoring Centre (2006), the use of the World Health Organization-Uppsala Monitoring Centre (WHO-UMC) system for standardised case causality assessment http://who-umc.org/graphics/4409.pdf.

[13] Vervloet D, Durham S (1998). ABC of allergies adverse reaction to drugs. Br Med J.

[14] Caranasos GJ, Stewart RB, Cluff LE (1974). Drug-induced illness leading to hospitalization. J Am Med Assn.

[15] Aellig HW (1998). Adverse reactions to antihypertensive therapy. Cardiovas Drug Ther.

[16] Lewis CE, Grandits GA, Flack J, McDonals R, Elmer PJ (1996). Efficacy and tolerance of antihypertensive treatment to men and women with stage-1 diastolic hypertension: Results of the treatment of mild hypertension study. Arch Intern Med.

[17] Montastruc JL, Lapeyre-Mestre M, Bagheri H, Fooladi A (2002). Gender differences in adverse drug reactions: Analysis of spontaneous reports to a regional pharmacovigilance centre in France. Fundam Clinical Pharmacol.

[18] Aqil M, Imam F, Hussain A, Alam MS, Kapur P, Pillai KK (2006). A pharmacovigilance study for monitoring adverse drug reactions with antihypertensive agents at a South Delhi hospital. Int J Pharm Pract.

[19] Sharma H, Aqil M, Imam F, Alam MS, Kapur P, Pillai KK (2007). A pharmacovigilance study in the department of medicine of a university teaching hospital. Pharmacy Pract.

[20] Goldberg AI, Dunley MC, Sweet CS (1995). Safety and Tolarability of losartan potassium, an angiotensine II receptor antagonist compared with hydrochlorthiazide, atenolol, felodipine ER and angiotensin converting enzyme inhibitors for the treatment of systemic hypertension. Am J Cardiol.

[21] Akici A, Kalaca S, Ugurlu U, Hale Z, Toklu, Oktay S (2007). Antihypertensive drug utilization at health centres in a district of Istanbul. Pharmacy World Sci.

